# The safety of magnetic resonance imaging contrast agents

**DOI:** 10.3389/ftox.2024.1376587

**Published:** 2024-08-12

**Authors:** Amy Cunningham, Martin Kirk, Emily Hong, Jing Yang, Tamara Howard, Adrian Brearley, Angelica Sáenz-Trevizo, Jacob Krawchuck, John Watt, Ian Henderson, Karol Dokladny, Joshua DeAguero, G. Patricia Escobar, Brent Wagner

**Affiliations:** ^1^ School of Medicine, University of New Mexico Health Science Center, Albuquerque, NM, United States; ^2^ Department of Chemistry and Chemical Biology, University of New Mexico, Albuquerque, NM, United States; ^3^ Cell Biology and Physiology, University of New Mexico Health Science Center, Albuquerque, NM, United States; ^4^ Department of Earth and Planetary Sciences, University of New Mexico, Albuquerque, NM, United States; ^5^ Sandia National Laboratory, Center for Integrated Nanotechnologies, Albuquerque, NM, United States; ^6^ Los Alamos National Laboratory, Center for Integrated Nanotechnologies, Albuquerque, NM, United States; ^7^ Omphalos Bioscience, Albuquerque, NM, United States; ^8^ Kidney Institute of New Mexico, University of New Mexico Health Science Center, Kidney Institute of New Mexico, Albuquerque, NM, United States; ^9^ New Mexico VA Healthcare System, Research Service, Albuquerque, NM, United States

**Keywords:** gadolinium, metals, gadodiamide, magnetic resonance imaging contrast, renal tubular epithelium, mitochondriopathy, electron microscopy, X-ray spectra

## Abstract

Gadolinium-based contrast agents are increasingly used in clinical practice. While these pharmaceuticals are verified causal agents in nephrogenic systemic fibrosis, there is a growing body of literature supporting their role as causal agents in symptoms associated with gadolinium exposure after intravenous use and encephalopathy following intrathecal administration. Gadolinium-based contrast agents are multidentate organic ligands that strongly bind the metal ion to reduce the toxicity of the metal. The notion that cationic gadolinium dissociates from these chelates and causes the disease is prevalent among patients and providers. We hypothesize that non-ligand-bound (soluble) gadolinium will be exceedingly low in patients. Soluble, ionic gadolinium is not likely to be the initial step in mediating any disease. The Kidney Institute of New Mexico was the first to identify gadolinium-rich nanoparticles in skin and kidney tissues from magnetic resonance imaging contrast agents in rodents. In 2023, they found similar nanoparticles in the kidney cells of humans with normal renal function, likely from contrast agents. We suspect these nanoparticles are the mediators of chronic toxicity from magnetic resonance imaging contrast agents. This article explores associations between gadolinium contrast and adverse health outcomes supported by clinical reports and rodent models.

## Introduction

**FIGURE 1:**
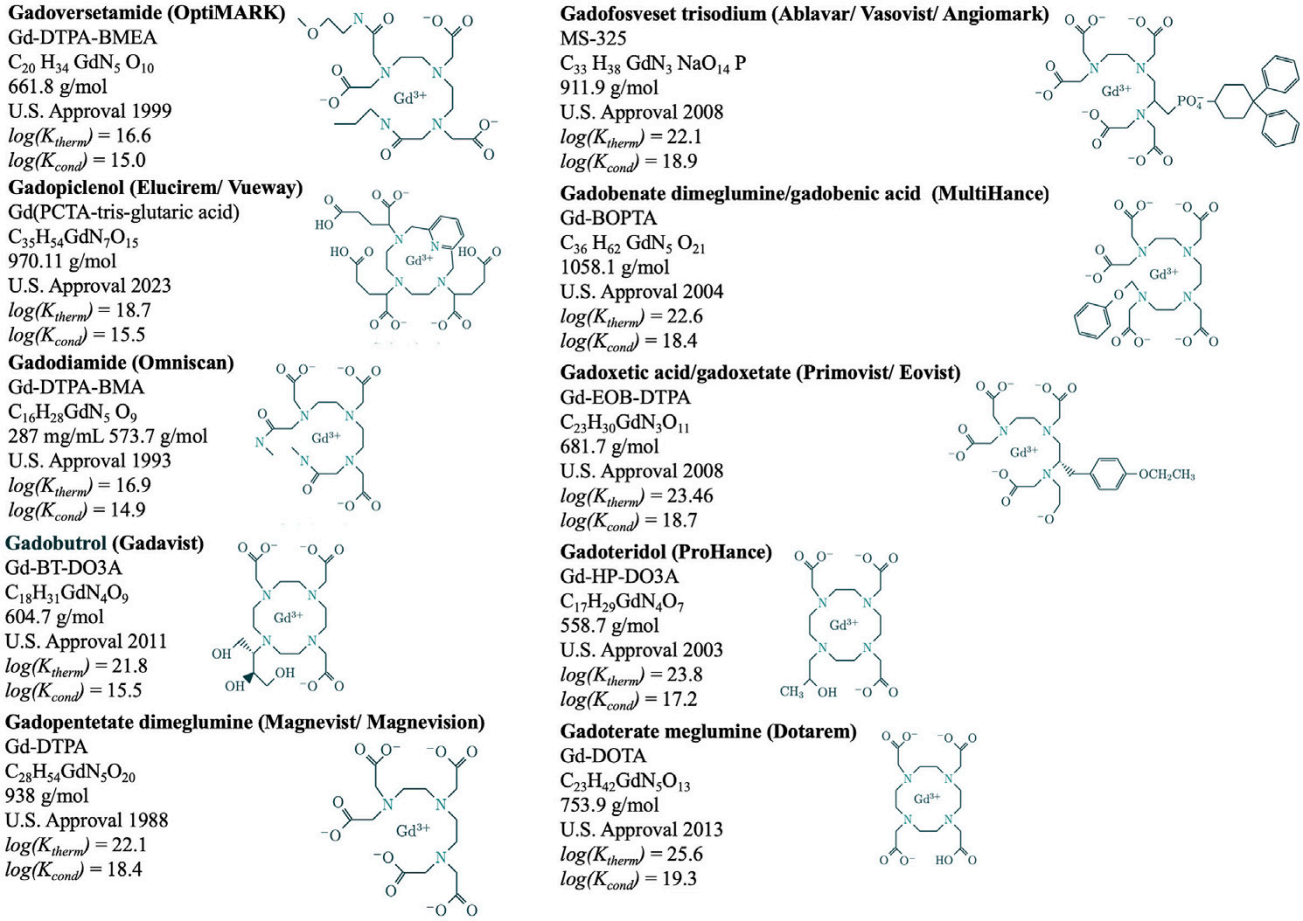
Magnetic resonance imaging contrast agent chemical structures, formulae, United States approval years, thermodynamic (*log(K*_*therm*_ *)*) and conditional [*log(K*_*cond*_ *)*] stability constants.

Rare earth metals are essential to contemporary technologies and comprise medicines (lanthanum) and intravenous contrast (gadolinium) applied to patients. Gadolinium is a toxic rare earth metal ideally suited to enhance magnetic resonance imaging scans. In late 2023, the National Center for Health Statistics introduced specific codes for magnetic resonance imaging contrast agent-induced toxicities for the International Classification of Diseases, 10th Edition, Clinical Modification (ICD-10-CM) (Table 1). Gadolinium-based contrast agents are multidentate organic ligands (Figure 1) that strongly bind the metal ion, reducing its toxicity. These contrast agents are eliminated in the urine, but detectable amounts of gadolinium still remain throughout the body (including the brain). Systemic fibrosis, kidney injury, and (occasionally fatal) encephalopathy are associated with exposure to gadolinium-based contrast agents (Wagner et al., 2012; Do et al., 2014; Wagner et al., 2016; Do et al., 2019a; Do et al., 2019b; Provenzano et al., 2019; Benzon et al., 2021; Bruno et al., 2021; Jackson et al., 2022). These associations are strong, consistent, temporal, plausible, and demonstrated with experiment.

**TABLE 1:** International classification of diseases, tenth edition, clinical modification (ICD-10-CM) diagnosis codes.^a^

Code	Description
T56	Toxic effects of metals
T56.82	Toxic effect of gadolinium
T56.821	Toxic effect of gadolinium, accidental/unintentional
T56.821A	… Initial encounter
T56.821D	… Subsequent encounter
T56.821S	… Sequela
T56.822	Toxic effect of gadolinium, intentional self-harm
T56.822A	… Initial encounter
T56.822D	… Subsequent encounter
T56.822S	… Sequela
T56.823	Toxic effect of gadolinium, assault
T56.823A	… Initial encounter
T56.823D	… Subsequent encounter
T56.823S	… Sequela
T56.824	Toxic effect of gadolinium, undetermined
T56.824A	… Initial encounter
T56.824D	… Subsequent encounter
T56.824S	… Sequela
S00-T88	Injury, poisoning and certain other consequences of external causes
T50.8X5	Adverse effect of diagnostic agents

^a^ The gadolinium-specific codes have been effective since 1 October 2023.

Most studies in nanotoxicology focus on the effects of nanoparticles generated from combustion and pollution, particularly within the environment. Hence, nanotoxicology publications focus on inhalation, ingestion, and skin exposures. Gadolinium-rich nanoparticles are unique in that they form internally after intravenous magnetic resonance imaging contrast agent administration (DeAguero et al., 2023). We have discovered that gadolinium is leached from the pharmaceutical ligands and amalgamated into spiculated nanoparticles (Do et al., 2019a; Do et al., 2019b; Do et al., 2020; DeAguero et al., 2023).

Currently, the quantification of the prevalence of symptoms associated with gadolinium-induced chronic toxicity presents a challenge. This difficulty stems primarily from the absence of a universally accepted clinical case definition for gadolinium toxicity. Criteria for “gadolinium deposition disease” (Ramalho et al., 2016; Semelka et al., 2016) have been proposed (Semelka and Ramalho, 2021; Semelka and Ramalho, 2023). Diagnosing and categorizing cases consistently becomes problematic without clear and standardized clinical criteria. The ICD-10-CM codes pertinent to gadolinium toxicity were introduced only a few months ago. This recent introduction means that historical medical data needs to include these specific codes, rendering longitudinal analyses and retrospective studies less effective in capturing the full scope of this condition. As a result, obtaining a reliable measure of the prevalence of symptoms related to gadolinium exposure is currently not feasible, underscoring a crucial gap in our understanding and hindering effective monitoring and response strategies.

### The causality of iatrogenic systemic fibrosis with magnetic resonance imaging contrast agents is indisputable for patients with acute or chronic kidney insufficiency

Safety concerns with magnetic resonance imaging contrast agents arose when gadolinium was linked to the blight systemic fibrosis, a grievous infirmity (Grobner, 2006). A new fibrotic dermal syndrome in patients with acute, chronic, and end-stage renal disease was first identified in 1997 by dermatologists in San Francisco (Cowper et al., 2000). This scleromyxoedema-like disease was detailed in nine transplant patients, five end-stage renal disease patients, and one patient with acute kidney injury from various cities (including, among others, San Francisco to Chicago and Atlanta). Unlike scleroderma, the disease primarily involved the extremities but spared the face. Unlike scleromyxedema, none of the patients had evidence of serum paraprotein. In addition to severe pain, the skin has been characterized as having a woody induration and cobblestoned. The disease is also associated with severely debilitating joint contractures.

### Systemic fibrosis is strongly associated with magnetic resonance imaging contrast agents

“Nephrogenic” systemic fibrosis is characterized by skin and subcutaneous thickening in addition to systemic manifestations, and gadolinium has been recovered from skin biopsies of patients suffering from systemic fibrosis (Birka et al., 2015). The association between systemic fibrosis and prior MRI contrast agent exposure was so strong it prompted an immediate response from drug regulating agencies (Wagner et al., 2016). By 2006, the United States Food & Drug Administration recommended that healthcare professionals weigh the benefits and the risks associated with gadolinium administration for patients with glomerular filtration rates less than 60 mL/min/1.73 m^2^ (FDA, 2006). The United States Food & Drug Administration required boxed warnings for gadolinium-based contrast agents in 2007. The warnings were the same regardless of contrast agent brand, noting that there was an increased risk of nephrogenic systemic fibrosis in patients with either acute or chronic impairment of glomerular filtration rates when exposed to gadolinium. The labeling changes for all marketed gadolinium-based contrast agents were aimed at describing and minimizing the risk of nephrogenic systemic fibrosis.

**FIGURE 2:**
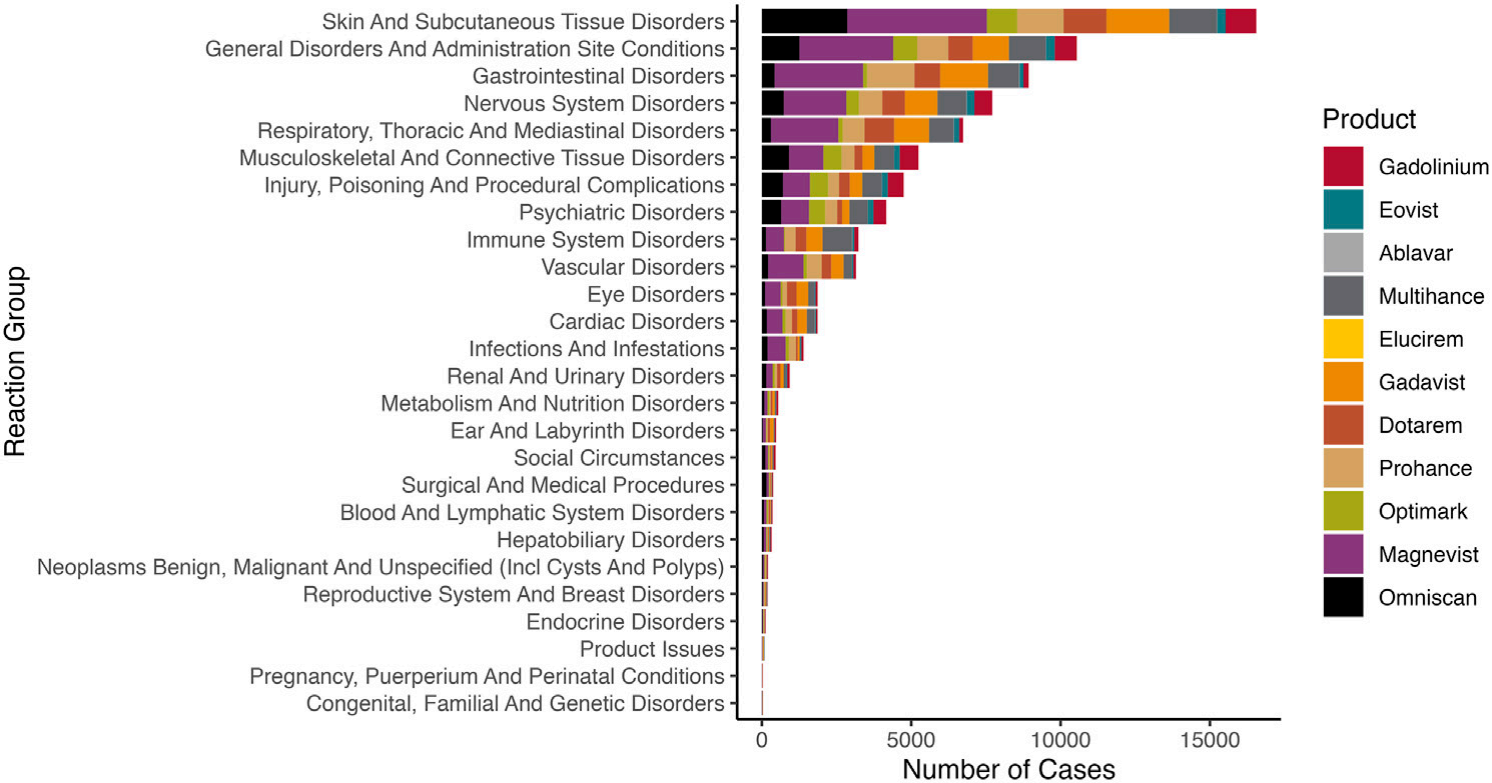
Reactions reported to the United States Food & Drug Administration Adverse Event Reporting System. We queried the FAERS Public Dashboard for magnetic resonance imaging contrast agents (generic and brand names).

Gadolinium-based contrast agents are increasingly associated with cutaneous and systemic abnormalities in patients with normal renal function. Gadolinium-based contrast agent-associated sclerotic plaques have been documented in patients with varying degrees of renal function without additional clinical features of nephrogenic systemic fibrosis (Gathings et al., 2015). A 2019 single-patient case report described a skin biopsy with deep dermal fibrosis, sclerotic bodies, and CD34 positivity consistent with a cutaneous reaction to gadolinium, which was confirmed present in the sample by inductively coupled plasma mass spectrometry (Olayiwola et al., 2019). Data mining of the United States Food & Drug Administration Adverse Event Reporting System corroborates that skin complications relate to most brands of magnetic resonance imaging contrast agents (Wang et al., 2023). As of 30 September 2023, 31,868 reactions were reported to the United States Food & Drug Administration Adverse Event Reporting System (Figure 2). The leading reaction group for all magnetic resonance imaging contrast agents is skin and subcutaneous tissue disorders (including nephrogenic systemic fibrosis).

### There is consistency between systemic fibrosis and different magnetic resonance imaging contrast agent exposure brands

**FIGURE 3:**
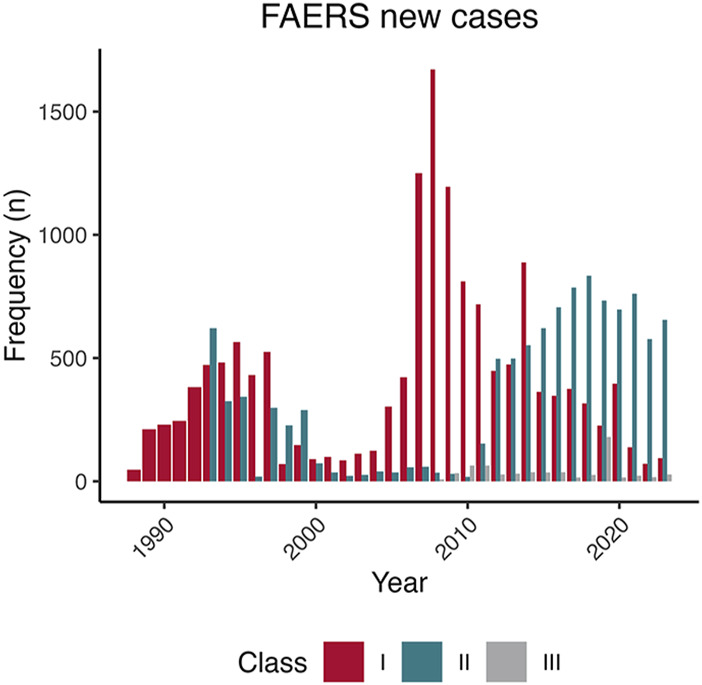
Unique cases were reported to the Food & Drug Administration Adverse Event Reporting System by the American College of Radiology Magnetic Resonance Imaging Contrast Agent Class. After discovering that gadolinium was the cause of nephrogenic systemic fibrosis, the American College of Radiology categorized magnetic resonance imaging contrast agents according to the number of cases associated with the complication at the time. Group I agents (Omniscan, OptiMark, and Magnevist) are presumed to have the highest association with nephrogenic systemic fibrosis, Group II agents (Dotarem, Gadavist, MultiHance, ProHance, and Vueway) are presumed to have less tendency to elicit nephrogenic systemic fibrosis. Group III agents (Ablavar, Eovist) have an unknown propensity to cause systemic fibrosis. Most Group II agents were approved after the association between gadolinium and systemic fibrosis was known. Because the market share of the only Group II agent, ProHance, was less than 5% before 2006, the propensity of this category and systemic fibrosis was likely in a window where there was more caution with their use (Leyba and Wagner, 2019).

There are many brands of gadolinium-based contrast agents. For example, Omniscan, Magnevist, OptiMARK, and Gadavist/Gadovist cause nephrogenic systemic fibrosis. The United States Food & Drug Administration Adverse Event Reporting System has reports of nephrogenic systemic fibrosis with MultiHance, ProHance, and Dotarem (Figure 3). There are published reports of nephrogenic systemic fibrosis with MultiHance (normal kidney function) and Dotarem. Nephrogenic systemic fibrosis is one of the top reactions for MultiHance listed in the United States Food & Drug Administration Adverse Event Reporting System. The United States Food & Drug Administration Adverse Event Reporting System case #11755699 (2015) details a nephrogenic systemic fibrosis-like reaction after two doses of MultiHance in the setting of normal kidney function. (Symptoms were skin thickening, “like hard rubber,” hair loss, skin biopsy with fibrosis, and abnormal calcification on mammograms and x-rays.) A 57-year-old woman exposed to MultiHance (plasma creatinine ranging from 0.9 to 1.2 mg/dL) developed edema and skin thickening of the lower extremities 3 months after exposure. Biopsy showed sclerosing dermopathy. The diagnosis was nephrogenic systemic fibrosis despite the estimated GFR > 60 mL/min/1.73 m^2^ at the time of administration (Lohani et al., 2017). The United States Food & Drug Administration Adverse Event Reporting System lists over 60 fibrosis cases with Dotarem. Dotarem caused nephrogenic systemic fibrosis in a 65-year-old man with a plasma creatinine of 1.28 mg/dL (Lim et al., 2020). Edema, erythema, joint contractures, and sclerosis manifested 185 days after Dotarem exposure.

The delayed manifestations imply a minuscule remnant concentration of gadolinium; a second event may be causative. These curiously long delays to presentations (when adequate renal clearance has been present) suggest that classical toxicologic mechanisms are not at play, but nanotoxicologic mechanisms are.

### Most patients with gadolinium-induced systemic fibrosis have magnetic resonance imaging contrast agent exposure histories, highlighting the specificity of the association

Nephrogenic systemic fibrosis may also be carried with solid organ transplants into recipients from gadolinium-exposed donors (Wahba et al., 2007; Scheinfeld, 2008). “[G]adolinium is commonly used in living liver organ donors to assess vasculature. Many transplant donors do not die of natural causes and are subjected to complex medical interventions before their deaths, which includes imaging with gadolinium” (Scheinfeld, 2008). Although under-recognized, nephrogenic systemic fibrosis is a specific histologic entity that is difficult to mistake.

### Gadolinium is temporally related to complications

Nephrogenic systemic fibrosis is temporally associated with gadolinium exposure (Khurana et al., 2007; Sadowski et al., 2007). The odds ratio for systemic fibrosis after gadolinium exposure is how we pinned systemic fibrosis on magnetic resonance imaging contrast agents (Wagner et al., 2016). Although retrospective clinical studies cannot provide an accurate incidence, studies have reported nephrogenic systemic fibrosis symptom onset as soon as a median of 11.5 days after gadodiamide exposure, ranging from 0.10–0.31 mmol/kg body weight (Marckmann et al., 2006; Sadowski et al., 2007). However, symptoms can take days to years to develop after gadolinium exposure (Schlaudecker and Bernheisel, 2009; Larson et al., 2015). In our experience, the diagnosis can be overlooked for years.

Acute kidney injury has also been temporally linked to gadolinium-based contrast administration. A retrospective analysis of 68 chronic kidney disease patients reported ten patients (14.7%) had developed kidney injury within 48 h of endovascular ProHance or Omniscan administration for renal stent placement (Takahashi et al., 2018). A review of 19 clinical studies reported acute kidney injury post-gadolinium contrast in 12%–50% of cases (Martino et al., 2021). Magnetic resonance imaging contrast agents at angiography doses are more nephrotoxic than iodinated (Rogosnitzky and Branch, 2016).

Neurotoxicity has been linked to magnetic resonance imaging contrast agents in rodent models and case reports (Rogosnitzky and Branch, 2016). Intrathecal gadolinium administration occurs as an inadvertent or off-label procedure due to a lack of robust trial data evaluating its use. However, formal studies have been pursued with doses up to 1.0 mL (Singh et al., 2016; Reeves et al., 2017). Encephalopathy has also been temporally linked to intrathecal gadolinium-based contrast administration. Intrathecal gadolinium may be administered off-label in fluoroscopic procedures in patients allergic to iodinated contrast. A 2012 case report described a patient who received approximately 1.0 mmol of epidural gadodiamide and experienced subsequent episodes of vomiting and altered mental status within 4 h of administration. She was diagnosed with encephalopathy (Moradian et al., 2022). The patient’s computerized tomography scans exhibited cerebral edema. Non-contrast brain magnetic resonance imaging showed hyperintensity in the sulci and ventricles consistent with gadolinium in the cerebrospinal fluid space (Moradian et al., 2022). A fatal encephalopathy case was reported in a patient receiving 5 mL epidural gadoteridol during a minimally invasive lumbar decompression (Provenzano et al., 2019). Postoperatively, the patient experienced a severe headache, altered mental status, apnea, agitation, and hypertonia of the arms and legs. Imaging showed gadolinium accumulation within the cerebrospinal fluid. The patient was diagnosed with encephalopathy secondary to intrathecal injection of gadolinium-based contrast (Provenzano et al., 2019).

### Do gadolinium complications imply the presence of biological gradients?

Known complications of gadolinium-based contrast agent administration include kidney damage (Leander et al., 1992; Prince et al., 1996; Sam et al., 2003; Thomsen, 2004; Akgun et al., 2006; Briguori et al., 2006; Ergun et al., 2006; Elmstahl et al., 2007), nephrogenic systemic fibrosis, skin disorders, and sometimes permanent neurologic sequelae (including coma and death). Each dose of gadolinium is fraught with unanticipated risks. Gadodiamide and gadoteridol significantly increase dermal cellularity in rodent models (Wagner et al., 2012, 2016). Quantitatively, the cellularity in rodent models is *identical* to that witnessed in patients with gadolinium-induced systemic fibrosis (Wagner et al., 2012; Nazarian et al., 2011). Dermal cellularity (measured in cells/high power field) quadrupled in the contrast agent-treated rats. The number of cells per high-power field was in the same order as reported in humans with nephrogenic systemic fibrosis attributed to Magnevist (Nazarian et al., 2011). The cells express myeloid markers, factor XIIIa, procollagen I, and the C-C chemokine receptor 2 (Wagner et al., 2012; Drel et al., 2016; Wagner et al., 2016; Do et al., 2019b; Bruno et al., 2021). Bone marrow has a memory of prior magnetic resonance imaging contrast agent exposure. We demonstrated that priming myeloid cells with a magnetic resonance contrast agent enhanced fibrotic response when lethally irradiated rats were challenged with gadolinium-based contrast agents (Drel et al., 2016). Myeloid memory explains why repeat exposures correlate with more severe disease manifestations.

In humans, *a single magnetic resonance imaging contrast agent exposure can trigger nephrogenic systemic fibrosis* (Broome et al., 2007; Thomsen et al., 2007; Abraham et al., 2008; Shabana et al., 2008; Leyba and Wagner, 2019). When Dr. Sean Cowper (Professor of Dermatology, Yale School of Medicine) maintained a registry of nephrogenic systemic fibrosis cases, he noted that 46% of cases had just a single exposure. For patients with end-stage renal disease, the odds ratios for systemic fibrosis after a single magnetic resonance imaging contrast agent dose range from 22 to 32.5 (Wagner et al., 2016). Manifestation of systemic fibrosis can occur *years after exposure*. A woman exposed to Magnevist in 2002 and then ProHance in 2005 began to manifest skin tightening, joint pain, and reduced range of motion in 2011 (Birka et al., 2015). In a retrospective analysis of end-stage renal disease patients in Scotland, the time from exposure to manifestation of nephrogenic systemic fibrosis ranged from 2 to 2395 days (Collidge et al., 2007). There was an association between the dose of contrast agent and nephrogenic systemic fibrosis.

*Magnetic resonance imaging contrast agents are not inert* (Wagner et al., 2012; Do et al., 2014; Drel et al., 2016; Wagner et al., 2016; Do et al., 2019a; Do et al., 2019b; Leyba and Wagner, 2019; Do et al., 2020; Bruno et al., 2021; Jackson et al., 2022; DeAguero et al., 2023; Wagner et al., 2023). Permanent gadolinium deposition is also a concern of some patients and providers. Since 2014, high-intensity signals on non-contrast-enhanced magnetic resonance images of the human brain have been attributed to gadolinium retention (Kanda et al., 2014). The signal intensity ratios in specific brain regions (dentate nucleus/pons, globus pallidus/thalamus) were most strongly related to the number of prior magnetic resonance imaging contrast agent exposures. Sometimes, radiologists can detect abnormal signal intensity in those with histories of just single magnetic resonance imaging contrast agent exposures (Benzon et al., 2021). While linear agent exposure often led to increased per-dose deposition, even one dose of a macrocyclic agent revealed noticeable brain tissue deposition in children (Stanescu et al., 2020). Triple-dosed contrast agent exposures over 1 year (Magnevist, n = 67 participants, 0.3 mmol/kg, 36.6 mean administrations) were correlated with bone retention (as assessed by hyperintense T1-weighted magnetic resonance imaging signal throughout the study). Adverse events from single administrations of gadolinium were described as ‘substantial’ at the 8 September 2017 United States Food & Drug Administration Medical Imaging Drugs Advisory Committee meeting (FDA, 2017).

*There are many reports of neurotoxicity induced by gadolinium* (Table 2). Many case reports detail acute, subacute, and chronic complications (summarized in Supplementary Material). Invariably, these cases required escalation of care for life-threatening scenarios. Common to these cases are instances when magnetic resonance imaging contrast agents have been introduced into the cerebrospinal fluid (intrathecal) compartment. The doses of magnetic resonance imaging contrast agent needed to induce severe neurologic manifestations (and sometimes death) are minute. The concentrations of gadolinium vary among formulations. Previous case reports have established that neurotoxic effects can be seen at gadolinium doses as low as 1 µmol/g brain tissue (Provenzano et al., 2019; Benzon et al., 2021). High-dose gadolinium can neurotoxically affect oligodendroglial and astroglial cells (Kapoor et al., 2010). Low intrathecal doses have resulted in multisystemic organ failure, coma, and death (Provenzano et al., 2019). These cases underline the potential neurotoxic effects of gadolinium, particularly when used intrathecally, and highlight the need for caution and further research into the safe use of magnetic resonance imaging contrast agents.

**TABLE 2:** Gadolinium-induced encephalopathy cases.^a^

Agent	Age	Sex	Dose (mmol)	Route	Outcome	Acute manifestations	Permanent complications	References
Gadavist	53	F	1	Intrathecal	Non-fatal	Headache, global aphasia, altered consciousness, tonic-clonic seizures	Anterograde amnesia	Palazón-Cabanes et al. (2016)
Gadavist	69	F	2	Intrathecal	Non-fatal	Agitation, vertigo, myoclonic jerks, aggression, asystole, sacral pain, fecal incontinence	Amnesia, impaired concentration and memory retention	Besteher et al. (2020)
Gadavist	73	F	2	Intrathecal	Non-fatal	Altered mental status, right gaze deviation, upper extremity tonicity, seizure-like posturing	NA	Platt et al. (2020)
Gadavist	NA	M	2	Intrathecal	Non-fatal	Headache, confusion, aphasia, generalized tonic-clonic seizures	NA	Chauhan and Upadhyay (2021)
Gadavist	60	F	2	Intrathecal	Non-fatal	Pain, spasms	NA	Reeves et al. (2017)
Omniscan	67	F	2	Intrathecal	Non-fatal	Disorientation, chills, dyspnea, nausea	NA	Samardzic and Thamburaj (2015)
Magnevist	42	M	3	Intrathecal	Non-fatal	Confusion, global aphasia, rigidity, seizures, myoclonus, neck stiffness, absent Babinski reflexes, hypertension, emesis	Visual disturbances	Park et al. (2010)
Omniscan	61	F	4	Intrathecal	Non-fatal	Mental status changes, grand mal seizure, respiratory failure	Intermittent partial seizure-like activity	Kapoor et al. (2010)
Omniscan	67	F	4	Epidural	Non-fatal	Altered mental status, attention deficits, hypoxia, tachycardia, hypertension, emesis	NA	Moradian et al. (2022)
Magnevist	59	M	5	Intrathecal	Non-fatal	Agitation, dysarthria, aphasia, depressed mentation, right facial droop, labile blood pressure, polyuria	Nonconvulsive status epilepticus	Nayak et al. (2013)
Magnevist	59	M	5	Intrathecal	Non-fatal	Aphasia, unilateral facial droop, delirium, nonconvulsive status epilepticus, nausea, hypertension	Non-communicative	Singh et al. (2016)
ProHance	67	F	5	Intrathecal	Fatal	Headache, mental status changes, agitation, myotonia, agitation, myoclonus, bradypnea, wide complex pulseless electrical activity, seizures	NA	Provenzano et al. (2019)
Magnevist	34	F	7.5	Intrathecal	Non-fatal	Headache, nausea, vomiting, coma, systemic seizures	NA	Li et al. (2008)
Gadavist	85	F	8	Intrathecal	Non-fatal	Agitation, lower extremity paresthesia, convulsive status epilepticus, nausea, pelvic pain	Residual cognitive impairment	Sutherland et al. (2021)
Gadavist	35	F	8	Intravenous	Fatal	Encephalopathy, absent cough/gag/corneal/deep tendon reflexes, dyspnea, respiratory distress, chest heaviness, tachycardia, hypotension, diarrhea, nausea, vomiting	NA	Chalise et al. (2023)
Magnevist	64	M	10	Intrathecal	Non-fatal	Confusion, nausea, vomiting, dysarthria, somnolence, blurred vision, delirium, ataxia, gaze-evoked nystagmus, disorientation, agitation, aggression, visual and auditory hallucinations, ataxia, acalculia	Concentration difficulties, gait ataxia	Arlt et al. (2007)
Gadavist	55	M	12	Intrathecal	Non-fatal	Nonverbality, convulsive and generalized seizures, coma, apnea, cyanosis, hemodynamic instability, nausea, emesis, diffuse weakness, areflexia	Severe anterograde memory deficits	Calvo et al. (2020)
NA	57	F	30–40	Intravenous	Non-fatal	Mental status changes	Retrograde amnesia	Maramattom et al. (2005)
Magnevist	55	F	NA	Intraventricular	Non-fatal	Headache, altered mental status	NA	Nayak et al. (2013)
NA	56	M	NA	Intrathecal	Non-fatal	Confusion, slurred speech, expressive aphasia, hypertension	NA	Pokersnik et al. (2018)

^a^ NA, not available; M, male; F, female.

## Discussion

The International Classification of Diseases, Tenth Revision, Clinical Modification (ICD-10-CM) added gadolinium-specific diagnostic codes in late 2023 (Table 1).

### There is evidence for the association between disease and magnetic resonance imaging contrast agent exposure

Regardless of brand, gadolinium-based contrast agents *consistently* have the highest incidences of nephrogenic systemic fibrosis, gadolinium deposition disease, skin diseases, and encephalopathies in the FDA Adverse Event Reporting System. As the FDA-approved prescribing information sheets attest, gadolinium is the cause of gadolinium-induced systemic fibrosis, gadolinium-induced kidney injury, and gadolinium encephalopathy. Gadolinium deposition is always anticipated with the intravenous administration of any gadolinium-based contrast agent. Often, there is a *temporal* relationship between magnetic resonance imaging contrast agent exposure and symptom onset. Even in cases of systemic fibrosis in contrast-naïve organ recipients, gadolinium is suspected to have been transferred by the solid organ (Wahba et al., 2007).

### It is plausible that rare earth metal exposure and retention leads to disease. The lanthanides are defined by having f-block electron orbitals, and these elements are physiologically alien in vertebrates

**FIGURE 4:**
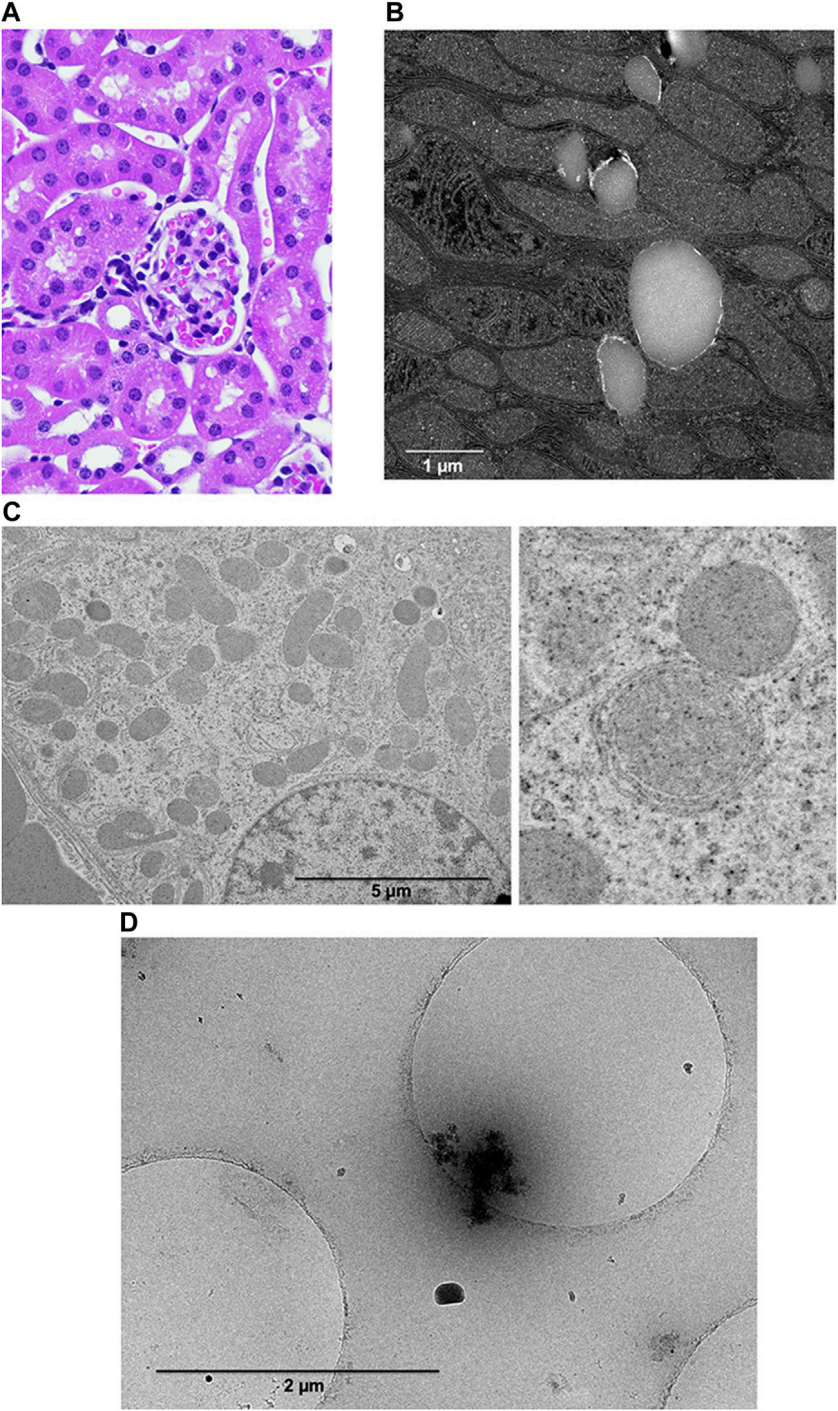
Systemic treatment with magnetic resonance imaging contrast agents induces the formation of gadolinium-rich nanoparticles. **(A)**. Renal cortex from magnetic resonance imaging contrast agent-treated mice may demonstrate subtle vacuolization by light microscopy. We obtain tissues 5 days or more after the last magnet resonance imaging contrast agent exposure. **(B)** Transmission scanning electron microscopy (darkfield mode) reveals unilamellar bodies rimmed with electron-dense material and mitochondrial swelling. FEI Tecnai G (2) S-Twin (300 kV) transmission electron microscope. **(C)** Transmission electron microscopy of a renal proximal tubular cell from a magnetic resonance imaging contrast-treated mouse. The shrunken, ballooned mitochondria are no longer oriented perpendicularly to the basolateral plane. The enlarged image demonstrates mitochondrial stress. **(D)** Cryo-transmission electron microscopy of nanoparticles from renal cortices from magnetic resonance imaging contrast agent-treated mice that were purified by ultracentrifugation through a sucrose gradient. The image shows a nanoparticle with an organic corona.

Case reports of neurologic damage (often permanent) and encephalopathy (sometimes fatal) incriminate small doses of gadolinium in profound biologic sequelae. The position that intracellular, foreign, rare earth metallic nanoparticles have no impact on cellular function is indefensible *prima facie*. Existing data suggests that nanotoxicity mediates complications induced by magnetic resonance imaging contrast agents (Figure 4).

Rodent studies demonstrate that magnetic resonance imaging contrast agents prime and activate bone marrow-derived circulating cells into pro-fibrotic states. Herein, we detail that gadolinium is neurotoxic. Gadolinium is detectable in the cerebrospinal fluid within minutes of magnetic resonance imaging contrast agent administration (Robert et al., 2018). The hypothesis that a physiologically alien rare earth metal causes many chronic symptoms is *coherent*.

### Experimental rodent models demonstrate analogous features to human disease

The markers we chose to investigate in the rodent models were entirely guided by what has been reported in human patients. CD34 is of critical importance as this marker stains wall-to-wall in human lesions. Factor XIIIa, procollagen I, CD45RO, and α-smooth muscle actin are all markers of fibrocytes, murine or human. In mice and humans, fibrosis is driven by TGF-β. Gadolinium-induced reactive oxygen species play a role in humans and rodents (Wagner et al., 2012, 2016; Drel et al., 2016). Dermal CD68-and CD163-positive macrophages are present in humans with gadolinium-induced systemic fibrosis and in rodent models (Drel et al., 2016; Do et al., 2019b; Wagner et al., 2012). Magnetic resonance imaging contrast agent degradation into intracellular nanoparticles is also an analogous feature with humans (DeAguero et al., 2023).

Nephrogenic systemic fibrosis has an established case definition (Leyba and Wagner, 2019). Rodent models demonstrate the exact mechanisms of fibrosis in human patients (Wagner et al., 2012; Do et al., 2014; Drel et al., 2016; Wagner et al., 2016; Do et al., 2019a; Do et al., 2019b; Do et al., 2020; Bruno et al., 2021; DeAguero et al., 2023). Dermal fibrosis is accompanied by many spindle-shaped cells, procollagen I, CD34, CD45RO, factor XIIIa, and α-smooth muscle actin stress fibers. In both mice and humans, fibrosis is driven by TGF-β.

Among metals, gadolinium is unique in inducing profound systemic fibrosis. There are similarities to other toxic metallic effects. Mercury can cause renal tubular injury. Lead can cause a range of acute and chronic effects. Cadmium is a nephrotoxic heavy metal that also induces fibrosis (Flores et al., 2011). Renal tubular dysfunction may exist (tubular proteinuria, glucosuria, aminoaciduria, hypercalciuria, impaired urinary concentration, and acid load impairment).

Contaminated rapeseed oil, tryptophan supplements, and bleomycin all induce skin fibrosis that mirrors specific characteristics of systemic sclerosis. In Spain, the ingestion of contaminated rapeseed oil led to a toxic oil syndrome, presenting with systemic sclerosis-like symptoms such as myalgia, neuropathy, and pulmonary hypertension (Tabuenca, 1981; Fonseca, 1983). Researchers have linked toxic oil syndrome to industrial oil altered with aniline. Despite fulfilling some Bradford Hill criteria—observing rapeseed oil with aniline derivatives among affected individuals—laboratory efforts to replicate the syndrome in animals failed, as aniline’s toxic effects did not align with those of toxic oil syndrome. The presence of nanoparticles in toxic oil syndrome remains unreported (Gelpi et al., 2002). The histopathology of toxic oil syndrome is similar to nephrogenic systemic fibrosis in that there is dermal fibrosis. In contrast, gadolinium-induced dermal fibrosis is characterized by cellular infiltration via monocyte chemoattractant-1 (Do et al., 2019a; Do et al., 2019b; Do et al., 2020; DeAguero et al., 2023) and its receptor, the C-C chemokine receptor 2, on CD34-positive circulating myeloid cells (Wagner et al., 2012; Drel et al., 2016; Wagner et al., 2016; Do et al., 2019a; Do et al., 2019b).

Similarly, the use of supplemental tryptophan has caused eosinophilia-myalgia syndrome, which shares features with nephrogenic systemic fibrosis, including chronic indurated skin, neuropathy, and myopathy. In these cases, systemic sclerosis autoantibodies are typically absent (Varga et al., 1990). Experimentally, researchers employ bleomycin to trigger pulmonary fibrosis, a condition closely related to scleroderma (Finch et al., 1980). Organic solvents (trichloroethane, trichloroethylene, toluene, xylene) have been linked to systemic sclerosis and systemic sclerosis-like syndromes (Nietert et al., 1998; Lacey et al., 1999; Maitre et al., 2004). These solvents, chemically akin to vinyl chloride, contribute to features typical of systemic sclerosis.

*Peau d’orange* appears in conditions such as inflammatory breast cancer, eosinophilic fasciitis, lymphatic obstruction (e.g., decompression illness), and systemic sclerosis. Histologically, dermal spindle cells mark some skin disorders. Dermatofibroma, dermatofibrosarcoma protuberans, Kaposi sarcoma, juvenile xanthogranuloma, and solitary fibrous tumors all show increased dermal cellularity. However, the specific risk factors for solitary fibrous tumors remain unclear. Spindle-shaped cells infiltrate the dermis in desmoplastic melanoma, but unlike in nephrogenic systemic fibrosis, these cells do not express CD34. Conversely, neurofibromas exhibit a high concentration of CD34-positive spindle cells (Yeh and McCalmont, 2011). Dermatofibrosarcoma protuberans and giant-cell fibroblastoma also present CD34-positive dermal spindle cells. Lesions from nephrogenic systemic fibrosis typically include factor XIIIa and CD68 (Jimenez et al., 2004; Kucher et al., 2005; Parsons et al., 2007; Bremmer et al., 2009; Wiedemeyer et al., 2009). In benign cephalic histiocytosis, the histiocytic infiltrate features dermal factor XIIIa and CD68.

**FIGURE 5:**
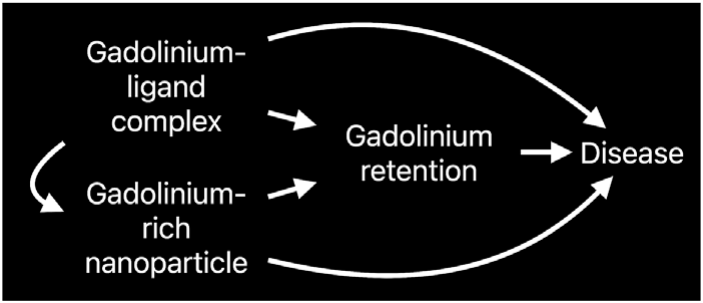
Causal diagram depicting the multiple avenues of metal-ligand complex-induced disease.

Conceptually, gadolinium-induced diseases may represent a continuum resulting from retaining a non-physiologic, toxic, heavy rare earth metal (Figure 5). Studies in animal models have shown the toxic effects of gadolinium in biological systems (Wagner et al., 2012; Drel et al., 2016; Wagner et al., 2016; Do et al., 2019a; Wagner, 2019; Do et al., 2020; Bruno et al., 2021; DeAguero et al., 2023). In humans, despite millions of exposures annually, magnetic resonance imaging contrast agents are generally well tolerated. The risk factors for catastrophic complications have yet to be elucidated. Patients with normal renal function develop similar or novel symptoms after gadolinium exposure. Gadolinium may be detectable in the blood, urine, and other tissues *years after exposure* to a magnetic resonance imaging contrast agent.

### It is unlikely that gadolinium (III) is found in the soluble state *in vivo* from magnetic resonance imaging contrast agent exposures

For the different pharmaceutical ligands, the affinities for the gadolinium cation in an equilibrium reaction strongly favor the chelated form (Eq. 1). The affinities for different types of gadolinium-ligand complexes are expressed in terms of ‘thermodynamic stabilities,’ *K*_*eq*_ (Eq. 2). These thermodynamic stabilities—defined in non-physiologic *in vitro* conditions—describe the propensity of the chelates to bind gadolinium.

$$Gd^{3+}+L^{3-}⇌GdL$$ (1)

$$\log\left(K_{eq}\right)=\frac{\left[GdL\right]}{\left[Gd\right]\left[L\right]}$$ (2)

Electron paramagnetic resonance (EPR) active *S* = 7/2 high-spin nature of Gd^3+^, coupled with fast exchange with water, makes Gd^3+^ a superior water relaxation catalyst for magnetic resonance imaging (Tweedle, 1997; Datta and Raymond, 2009). However, the high toxicity of Gd^3+^ requires this ion to be tightly bound by multidentate chelating agents (*L*) when functioning in commercial magnetic resonance imaging contrast agents. However, with *log* (*K*_*eq*_) values between 16–25, unassisted spontaneous dechelation to yield soluble (‘free’) Gd^3+^ represents an unlikely event (Hao et al., 2012). Ligand exchange with solvent water occurs via an associative mechanism (Datta and Raymond, 2009), and an initial equilibrium is present with a fast water exchange rate (Eq. 3),

$$Gd{\left(H_{2}O\right)}_{8}+L\;⇌Gd\left[\kappa^{7}-L\right]\left(H_{2}O\right)$$ (3)

Wherein *k* ^*7*^ *-L* denotes a 7-dentate ligand. In the presence of an alternative tight binding ligand (*e.g.,* oxalate, a bidentate ligand, *X*), we can imagine the simple equilibrium below:

$$Gd\left[\kappa^{7}-L\right]\left(H_{2}O\right)+X\;⇌Gd\left[\kappa^{5}-L\right]\left[\kappa^{2}-X\right]\left(H_{2}O\right)$$ (4)

Alternatively, *X* could be imagined displacing water, leading to the equilibrium:

$$Gd\left[\kappa^{7}-L\right]\left(H_{2}O\right)+X\;⇌Gd\left[\kappa^{6}-L\right]\left[\kappa^{2}-X\right]+H_{2}O$$ (5)

Provided *X* binds more tightly than the chelate, the equilibrium will lie on the right-hand side of the equation. The initial step in assisted dechelation is binding *X* to gadolinium (Hao et al., 2012), whereby additional binding of *X* ligands can lead to precipitation (Eq. 6).

$$2Gd\left[\kappa^{5}-L\right]\left[\kappa^{2}-X\right]\left(H_{2}O\right)+X+8H_{2}O⇌{Gd}_{2}{\left[\kappa^{2}-X\right]}_{3}{\left(H_{2}O\right)}_{10}↓+2L$$ (6)

The initial substitution reactions described by Eqs 4, 5 could occur by various means, including dissociative mechanisms. For example, oxalate may serve as a surrogate *in vitro* entering ligand. The reaction will form Gd_2_ (oxalate)_3_(H_2_O)_10_, which is insoluble and precipitates from solution. Gadolinium is effectively driven out of the chelate, potentially quite rapidly. An analogous reaction *in vivo* would be the complexation of the contrast agent with any physiological reactant that can bind strongly to *Gd(κ* ^*7*^ *-L)(H*_*2*_ *O)*. Any process that removes gadolinium from the solution shifts this equilibrium to the right via *Le Châtelier’s* principle and decreases the concentration of the metallated gadolinium-based contrast agent, *Gd(κ* ^*7*^ *-L)(H*_*2*_ *O)*. Notably, the kinetics of the ligand exchange reactions are important. However, soluble (“free”) Gd^3+^ concentration is likely to be unimportant in *in vivo* toxicity, and the kinetics of the exchange reaction with exogenous ligands are critical to delivering Gd^3+^ to the tissue where it is rendered insoluble.

### Magnetic resonance imaging contrast agents undergo degradation in patients, transforming into toxic, electron-dense, intracellular nanoparticles

We detected gadolinium-rich nanoparticles formed from systemic treatment with magnetic resonance imaging contrast agents (Do et al., 2019a; Do et al., 2019b; Do et al., 2020; DeAguero et al., 2023). We can process specimens like skin or kidneys with or without staining for transmission electron microscopy. We discovered electron-dense intracellular nanoparticles in the skin and kidney from the contrast agent-treated groups. We use multiple methods (X-ray energy dispersive and electron energy loss spectroscopy) to confirm that these intracellular sediments contain gadolinium. We have robustly confirmed these data using various instruments at multiple institutions: the University of Texas San Antonio (Hitachi SEM1510 scanning electron microscope), the University of New Mexico (JEOL NEOARM 200 kV aberration-corrected high-resolution scanning/transmission electron microscope equipped with two 100 mm2 energy dispersive spectroscopy detectors and JEOL 2010F FEGSTEM 200 kV), the University of New Mexico Health Sciences Center (Hitachi HT7700 transmission electron microscope), and the Center for Integrated Nanotechnologies (FEI Tecnai G (2) F30 S-Twin 300 kV with Fischione Instruments HAADF STEM detectors).

Therefore, physiologic conditions *substantially modify* the chemical equilibration, lending to the dissociation of gadolinium from the ligand (Eq. 3). In the presence of intracellular gadolinium-rich deposits, gadolinium continues to dissociate from the proprietary chelates. Because the dissociated gadolinium is trapped in insoluble nanoparticles, gadolinium is effectively out-of-play for a reverse reaction (i.e., *Gd(κ* ^*7*^ *-L)(H*_*2*_ *O)*). This application of *Le Châtelier’s* principle, combined with the modified equilibrium due to an *in vivo* environment, means that gadolinium will disassociate from the pharmaceutical contrast no matter the brand of contrast agent (regardless of the *log(K*_*eq*_ *)*). Intracellular rare earth metallic nanoparticles indisputably disrupt cellular harmonics. The ability of gadolinium to de-chelate has significant implications for current diagnostic practices and initial and repeated exposures. Intracellular, intraneuronal gadolinium-laden debris should not be subject to elimination by chelates relegated to the extracellular space.

There is no consensus on the diagnostic criteria for symptoms associated with gadolinium exposure/gadolinium deposition disease (Davies et al., 2022; McDonald et al., 2022). Hence, no evidence-based therapies exist for people with chronic symptoms attributed to magnetic resonance imaging contrast agent exposure. Patients with complications from contrast are migrating to providers giving untested therapeutics such as chelation—diethylenetriamine pentaacetate (DTPA) and ethylenediamine tetraacetic acid (EDTA) (Semelka et al., 2018). The pharmaceutical ligands comprising gadolinium-based contrast agents are grounded in the DTPA affinity for rare earth metals. Given the lack of accepted clinical criteria for gadolinium toxicity (McDonald et al., 2022), evidence for chelation therapy is scant (Lyapustina et al., 2019). The on-label indication for Ca-DTPA is concrete, i.e., to enhance the elimination of plutonium, americium, or curium when internal contamination with these transuranic elements has been within 24 h. Depletion of physiologic metals by chelation carries teratogenic and embryotoxic risks. The prescribing information sheet for Ca-DTPA notes that deaths have occurred in patients with hemochromatosis. Furthermore, repeat administrations of Ca-DTPA depleted zinc and manganese from the small intestine, skeleton, pancreas, and testes.

The scientific basis of how chelation therapy can target minute quantities of gadolinium in a clinically significant manner needs elucidation. To date, of participants in our study (n = 55) with histories of magnetic resonance imaging contrast agent exposure (10 days–15.5 years after the latest administration), 70% have serum gadolinium concentrations less than 640 p.m. Concentrations of physiologic cations, including transition metals, dwarf the detection limit of gadolinium: Calcium by 32,000,000 fold, copper by 195,000 fold, zinc by 261,000 fold, and manganese by more than 17 fold. DTPA and EDTA chelate these physiologic cations as well. There are risks of chelation therapy (Risher and Amler, 2005; Brown et al., 2006; American College of Medical T, 2010; Davies et al., 2022). Objective prospective clinical trials of chelation therapy for gadolinium are needed (Lyapustina et al., 2019; Davies et al., 2022).

### Intracellular gadolinium-rich nanoparticles may explain individual patients’ symptom variability and risk thresholds

Nanotoxicology is in its infancy (Elsaesser and Howard, 2012). Before the industrial age, humans were not exposed to manufactured nanoparticles less than 50 nm in diameter. Now, nanomaterials are generated on industrial scales, including for medicine. Nanotoxicity differs from conventional toxins and toxicants in that nanoscale materials' surface chemistry and chemical reactivities vary from the components (Elsaesser and Howard, 2012). Nanoparticles have multiple properties: size, shape, density, charge, and composition—novel and unique physiochemical properties that are not biologically inert (Nel et al., 2006). Intracellularly, a nanoparticle’s surrounding material – the corona – imparts many toxicologic effects.

**FIGURE 6:**
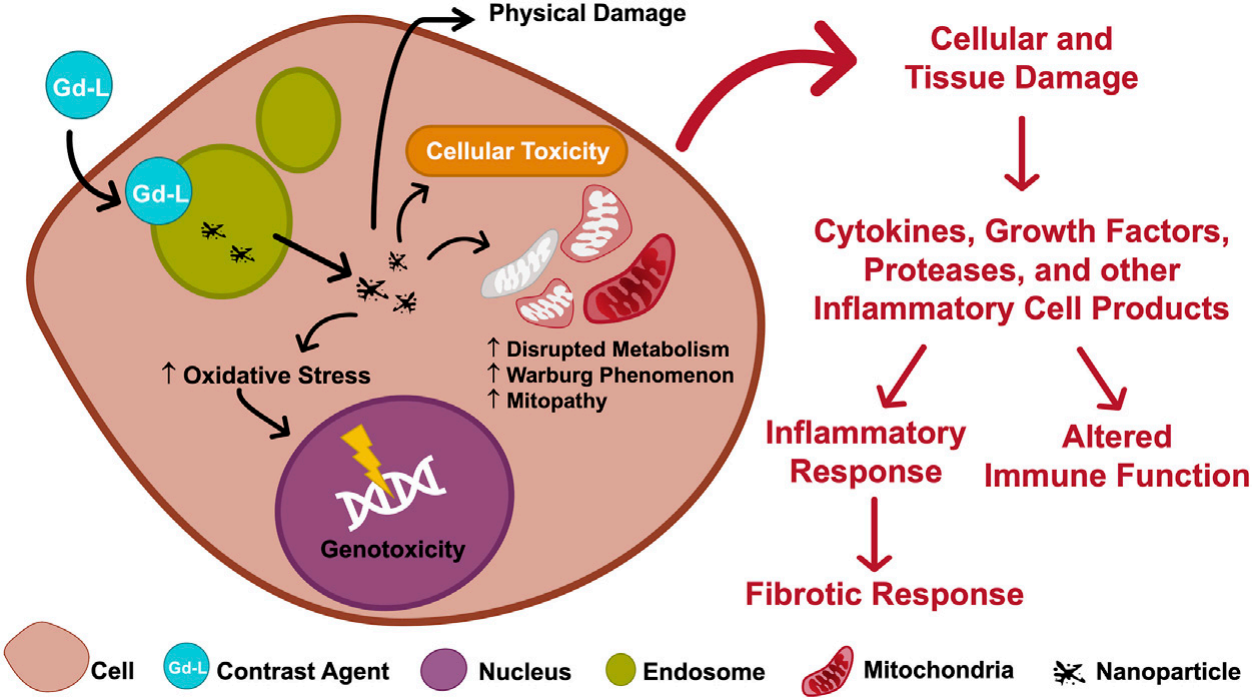
Mechanistic framework linking gadolinium retention to disease.

The principle of the Trojan Horse is that nanoparticle cellular internalization initiates toxic effects Krug and Wick (2011). Magnetic resonance imaging contrast agents have the potential for noxious effects (Pasquini et al., 2018). Nephrogenic systemic fibrosis, acute kidney injury, and encephalopathy are incontestable complications of magnetic resonance imaging contrast agents. Physiologically, gadolinium is a *sui generis* metal. The degradation of magnetic resonance imaging contrast agents into gadolinium-rich nanoparticles may be the initial step in complications and chronic disease. The nanoparticulate form of gadolinium differs from the base element gadolinium. Several experimental effects of magnetic resonance imaging contrast agent-induced gadolinium-rich nanoparticles include reactive oxygen species generation, oxidative stress (Wagner et al., 2012; Drel et al., 2016; Wagner et al., 2016; Bruno et al., 2021), mitochondrial perturbation (DeAguero et al., 2023) (Figure 6), tissue infiltration with inflammatory cells (Wagner et al., 2012; Drel et al., 2016; Wagner et al., 2016; Do et al., 2019a; Do et al., 2019b; Do et al., 2022), and uptake in brain neurons (McDonald et al., 2017; Stanescu et al., 2020; Davies et al., 2021). Our data makes us suspect that the nanoparticles are forming within the cells.

A fundamental principle of conventional toxicology is that effects are determined by dose and duration of exposure (Merugu et al., 2022). Magnetic resonance imaging contrast agents cause systemic fibrosis. Repeat administrations of magnetic resonance imaging contrast agents lead to permanent retention of gadolinium in the brain (DeBevits et al., 2020; Bageac et al., 2021). As previously reviewed, both linear and macrocyclic agents were found in post-mortem human brain specimens from patients with normal renal function. However, concentrations of deposited macrocyclic agents were comparatively lower than with linear agents (Ramalho et al., 2017). The implications of harboring potentially toxic nanoparticles in organs such as the brain have yet to be thoroughly studied.

*Risk is the product of severity and probability.* Although systemic fibrosis and encephalopathy are rare, they can be catastrophic. More often, acute reactions are reported with magnetic resonance imaging contrast agents. Chemical structures can categorize magnetic resonance imaging contrast agents (Figure 1). In an analysis of 158,100 patients and 281,945 magnetic resonance imaging contrast agent injections at the Mayo Clinic, Omniscan (a linear, American College of Radiology group I agent) had the lowest rate of allergic-like and physiological reactions when compared to Gadavist, MultiHance, and ProHance (i.e., the American College of Radiology group II agents) (McDonald et al., 2019). A systematic review found the lowest rate of immediate reactions with Omniscan, followed by Magnevist, Dotarem, Gadavist, ProHance, MultiHance, Primovist, and Ablavar (Behzadi et al., 2018). The authors concluded that protein binding, ionicity, and macrocyclic structures were associated with the highest rates of allergic-like adverse events.

### There is more than one side to the argument concerning the safety of magnetic resonance imaging contrast agents

Disinherited by the medical establishment, patients spend an eternal time in chronic symptomatic purgatory. Escaping complications from a gadolinium administration is not a sign of providence. It indicates that there are undiscovered factors. There are many knowledge gaps concerning magnetic resonance imaging contrast agents, the biology of gadolinium, and the mechanisms of complications. There is a lack of effective treatments for iatrogenic gadolinium metallosis. Clinicians should be aware of the possibility of gadolinium toxicity even in patients with normal renal function, especially with intrathecal administration. Neurological symptoms following intrathecal exposure can be transient, resolving with attentive medical management, but can result in severe and potentially permanent complications. More research is needed to find ways to prevent or slow the progression of these diseases.

Magnetic resonance imaging contrast agents cause kidney injury and gadolinium encephalopathy (sometimes fatal) and may lead to permanent gadolinium retention. Provider education regarding these known adverse events is critical, and informing patients of these risks and outcomes is essential.

## Data Availability

Publicly available datasets were analyzed in this study. These data can be found here: https://www.fda.gov/drugs/questions-and-answers-fdas-adverse-event-reporting-system-faers/fda-adverse-event-reporting-system-faers-public-dashboard.
