# Supplementary material for: The safety of magnetic resonance imaging contrast agents
Source: Front Toxicol. 2024 Aug 12;6:1376587. doi: 10.3389/ftox.2024.1376587 (PMC11345262; doi:10.3389/ftox.2024.1376587)
Supplement: Supplementary file 1 [file Table1.docx]

Supplementary Material

# Supplementary Data: Case reports.

Li et al. (1) documented a 34-year-old woman receiving 7.5 mmol (5.35 umol/g brain tissue) of intrathecal gadopentetic acid during myelography following surgical correction of a traumatic brachial plexus injury. The patient experienced immediate onset headache, nausea, and vomiting, becoming comatose approximately 1-hour post-injection, and developed systemic seizures. Follow-up magnetic resonance imaging on day six post-injection showed severe cerebral artery vasospasm, restricted diffusion in various brain areas, and Gd-DTPA accumulation, leading to hyper-osmolarity of the extracellular space. The patient recovered without significant complaints at 6- and 8-month follow-up visits. The article suggested that intrathecal gadolinium should be diluted with the patient’s native cerebrospinal fluid (1:5 to 1:10) before injection and with slow dose administration (approximately 1mL/min) to reduce the risk of hyperosmolar toxic effects.

Maramattom et al. (2) reported a 57-year-old woman in renal failure who developed a subacute encephalopathy after inadvertently receiving repeated gadolinium contrast administration during alternate-day magnetic resonance imaging procedures and magnetic resonance angiograms of the brain. The total dose was 60 – 80 mL of gadolinium-based contrast agent of unspecified name or concentration. The patient’s mental status declined progressively over a week, and the magnetic resonance images revealed “multiple subcortical hyperintensities on T2-weighted and fluid-attenuated inversion recovery images (multiple lacunar infarcts) without changes of a posterior encephalopathy syndrome.” The patient’s cerebrospinal fluid became “increasingly hyperintense on successive MRI scans,” indicating gadolinium diffusion into the cerebrospinal fluid. The serum-free gadolinium was 28,591 ng/mL on day 14 post-exposure. The patient’s mental status improved, which “coincided with the expected clearance of serum gadolinium and resolution of cerebrospinal fluid hyperintensity.” The authors thus hypothesized that this patient experienced gadolinium-induced encephalopathy due to gadolinium retention for more than two weeks after the last dose of contrast.

Provenzano et al. (3) described a 67-year-old woman who developed gadolinium encephalopathy following inadvertent intrathecal administration of an unspecified amount of gadoteridol for two fluoroscopically-guided minimally invasive lumbar decompression procedures. The patient immediately experienced “a severe headache, mental status changes, apnea, agitation, and increased muscle tone in arms and legs” while in the recovery room and was transferred to a hospital. The patient demonstrated eye and tongue twitching, thrashing, cries, decreased respiration, and myoclonic activity, followed by wide-complex pulseless tachycardia. After cardiopulmonary resuscitation, the patient “experienced a fever of 103°F (39.4°C) that did not resolve with acetaminophen and ice packs” and seizures approximately every 20 minutes. It was determined “it was too late to drain the patient’s [cerebrospinal fluid].” A review of computed tomography images from postoperative day 0 demonstrated “hyperattenuation in the basal cisterns, third ventricle, lateral ventricles, and right Sylvian fissure.” Thoracic spinal computed tomography on postoperative day 0 revealed gadolinium in the intrathecal space. Computed tomography and magnetic resonance imaging from postoperative day 4 showed suspected “gadolinium-induced cytotoxic edema in the right parietal lobe” and bilateral occipital region and infarctions suggestive of anoxic brain injury. Two weeks following the minimally invasive lumbar decompression procedure, the patient succumbed to multiorgan system failure. The autopsy revealed large cortical infarcts in the right occipital and parietal lobes, ischemic cerebellar changes, and mild gliosis of the hippocampus and lower pons with no evidence of subarachnoid hemorrhage. The cause of death was attributed to gadolinium neurotoxicity and hypoxic-ischemic encephalopathy due to cardiopulmonary arrest.

Samardzic et al. (4) documented a 67-year-old woman who developed gadolinium encephalopathy after inadvertent intrathecal delivery of 2 mmol Omniscan for a fluoroscopically-guided epidural steroid injection. Approximately 3 hours post-administration, she presented to an emergency department with nausea, dyspnea, and subjective chills. She subsequently deteriorated into a disoriented state, “disoriented to place and time but without focal deficits.” Non-enhanced brain magnetic resonance imaging revealed characteristic features of intrathecal gadolinium, including a pseudo-T2 appearance on T1-weighted images. Gadolinium appeared to have diffused into “the brain parenchyma, olivary bodies, and the membranous labyrinth.” A nulled cerebrospinal fluid signal was absent on fluid attenuation recovery imaging. The susceptibility-weighted imaging features resembled a subarachnoid hemorrhage. Computed tomography images showed a “pseudo-cerebral edema pattern given the high attenuation characteristics of gadolinium.”

Besteher et al. (5) described a 69-year-old woman with acute onset encephalopathy and cardiac arrest following diagnostic intrathecal administration of gadobutrol. The patient received 2 mmol gadobutrol to identify the site of a cerebrospinal fluid leak. Fifteen minutes after injection, the patient developed severe progressive agitation, physical aggressiveness, massive sacral pain, vertigo, myoclonic jerks, disorientation, nausea, vomiting, and uncontrolled defecation. Cerebral and spinal magnetic resonance imaging approximately 4 hours after gadolinium exposure revealed contrast “enhancement of the frontobasal cortex and surface of the brain stem” and contrast “distributed throughout the spinal and intracranial [cerebrospinal fluid] compartments.” The patient developed cardiac arrhythmias and asystolic episodes of 5-15 seconds after extubation. Intensive treatment stabilized the patient within a week of intrathecal gadolinium exposure. After four weeks, though the patient reported improved mood, cognitive difficulties remained, including disturbed concentration, retentiveness, and “amnesia for the day after gadolinium injection.”

Platt et al. (6) reported a 73-year-old woman presented with altered mental status one day after receiving 2 mmol gadobutrol for a lumbar epidural steroid injection complicated by a dural puncture. The patient also acutely exhibited right gaze deviation and tonic posturing of the upper extremities. Non-contrast computed tomography on admission revealed diffuse subarachnoid hyperdensity resembling a hemorrhage. Magnetic resonance imaging showed diffuse cerebrospinal fluid opacities consistent with gadolinium artifacts. Similarly, a computed tomography scan of the lumbar spine showed persistent hyperdensities within the thecal sac. The patient ultimately recovered from a formal diagnosis of pseudo-subarachnoid hemorrhage and gadolinium encephalopathy.

Kapoor et al. (7) published a case of a 61-year-old woman who received two doses of Omniscan (4 mmol each) within approximately 4 hours. Providers administered an initial gadolinium dose for a fluoroscopically-guided epidural steroid injection. Within 30 minutes of administration, she “developed a postdural puncture headache.” Symptoms persisted despite intravenous fluid administration. An epidural autologous blood patch was performed with another 4 mmol epidural gadolinium dose. Although the patient’s headache substantially subsided, she then suffered a grand-mal seizure and mental status changes. A head computed tomography scan and a non-contrast magnetic resonance imaging showed residual gadolinium and subarachnoid hyperintensities in the following days. Fluid attenuated inversion recovery) /T1-weighted magnetic resonance imaging images without contrast showed nearly resolved gadolinium accumulation six days after admission. The patient recovered by day 10. At six months post-discharge, the patient reported having intermittent partial seizure-like activity controlled with phenytoin.

Calvo et al. (8) reported a 55-year-old man who developed nausea, emesis, aphony, and convulsive seizures within minutes of inadvertent intrathecal administration of 12 mmol gadobutrol for cervical myelography. He became apneic, cyanotic, and hemodynamically unstable. He was comatose, lacking “cold caloric, gag/cough, and deep tendon reflexes." Initial computed tomography images demonstrated “diffuse contrast enhancement of subarachnoid, cisternal, and intraventricular compartments.” While “brainstem reflexes normalized within 12 hours of [gadolinium] exposure,” status epilepticus persisted for nine days. Day 8 magnetic resonance imaging showed continued diffuse subarachnoid and cisternal contrast enhancement. On day 11, the patient regained consciousness, yet still with “encephalopathy… diffuse weakness… and areflexia.” After three weeks, his neurologic status gradually improved. At his 25-week follow-up, the patient reported anterograde memory deficits but had not experienced subsequent seizures, and encephalopathy had improved. Parenchymal gadolinium deposition was notable 23 days after exposure by an unenhanced magnetic resonance imaging scan.

A 64-year-old man experienced encephalopathic symptoms after inadvertent intrathecal administration of 10 mmol Magnevist (9). Providers administered an additional 20 ml of iodine to complete the procedure once they discovered the error. Soon after that, the patient was confused with nausea and repeated vomiting. Within an hour, the patient became somnolent with progressive dysarthria and blurred vision. Magnetic resonance imaging obtained approximately 1 hour after gadolinium exposure showed subarachnoid contrast enhancement. Within three hours, the patient experienced delirium with “somnolence… limb ataxia, and gaze-evoked nystagmus.” By the time he was transferred to a neurocritical care unit, he was neither speaking nor responding to verbal commands. Routine labs were unremarkable. Over the next four days, the patient regained alertness but remained disoriented, restless, and aggressive, with mild upper limb ataxia and visual and auditory hallucinations. Occipital subarachnoid enhancement was present, attributed to the patient’s supine position. Neurological symptoms gradually recovered through day ten. He was discharged with mild residual gait and limb ataxia. The patient retrospectively reported partial anterograde amnesia for five days during this hospital admission. On follow-up 56 days after gadolinium exposure, the patient exhibited mild gait ataxia and reported concentration difficulties. No subarachnoid gadolinium was detected on the follow-up magnetic resonance imaging. Serial serum and cerebrospinal fluid gadolinium measurements from 10 hours to 5 days after administration showed a gradual decline from 23,365 nmol/mL in cerebrospinal fluid and 22.5 nmol/mL in serum to 117 nmol/mL in cerebrospinal fluid and undetectable levels in serum.

Moradian et al. (10) documented a 67-year-old woman who received inadvertent intrathecal gadodiamide (estimated dose of 1 mmol) for an L5-S1 epidural steroid injection. The patient vomited two and four hours after administration. She developed altered mental status 4 hours after exposure. The patient subsequently presented to their local emergency department. She was tachycardic, hypertensive, and hypoxic, with poor attention but no focal neurological deficits. A head computed tomography scan showed “diffuse cerebral edema with effacement of sulci.” The patient was diagnosed with encephalopathy and transferred to a tertiary care center for management. On admission, T1 magnetic resonance imaging and fluid-attenuated inversion recovery images without contrast showed hyperintense sulci and ventricles consistent with residual gadolinium in the cerebrospinal fluid. Electroencephalography confirmed encephalopathy. The patient was diagnosed with intrathecal gadolinium encephalopathy “due to the temporal association between the patient’s symptoms and intrathecal injection of gadolinium, neuro-imaging findings confirming the presence of gadolinium with the CSF spaces, and the lack of any other laboratory abnormalities and alternative conditions.” The patient recovered within hours of intravenous dexamethasone administration and was discharged. The patient recovered without sequelae at one- and six-month follow-up visits. Repeated magnetic resonance imaging five months post-injection showed resolution of cerebrospinal fluid hyperintensity.

A 56-year-old man experienced confusion, slurred speech, hypertension, expressive aphasia, and an otherwise unremarkable physical exam approximately 3 hours after receiving gadolinium for evaluation of an intrathecal pain pump (11). Non-contrast brain computed tomography imaging showed diffusely attenuated signals in the basal cisterns, and the patient was diagnosed with gadolinium encephalopathy. Care necessitated transfer to a neurological intensive care unit, where the man regained baseline neurological function and was discharged home six days after exposure.

Sutherland et al. (12) described an 85-year-old woman who received 8 mmol intrathecal Gadavist during a computed tomography myelogram. Within 10 minutes, the patient suffered “abrupt onset pelvic pain, nausea, and bilateral lower limb paresthesia.” The patient rapidly deteriorated and experienced extreme agitation and convulsive status epilepticus, confirmed by electroencephalography, which normalized 24 days later. “The patient was discharged to a rehabilitation hospital with moderate residual cognitive impairments.”

A 53-year-old woman received 1 mmol gadobutrol in the subarachnoid space during a magnetic resonance cisternography procedure to examine suspected cerebrospinal fluid leakage (13). Five hours afterward, the patient suffered “headache, fluctuating global aphasia… deterioration of consciousness, and generalized tonic-clonic seizures.” Cranial computed tomography revealed diffuse subarachnoid hyperdensity and “bubbles compatible with pneumoencephalus in the subarachnoid and intraventricular spaces.” The patient’s symptoms resolved within 24 hours, though the patient experienced transient anterograde amnesia. Providers ruled out subarachnoid hemorrhage and infectious meningitis with a lumbar puncture. The patient was diagnosed with encephalopathy due to intrathecal gadolinium exposure.

Chauhan et al. (14) documented a patient of unspecified age and sex who received two mmol of intrathecal gadobutrol during a lumbar epidural steroid injection. Within 30 minutes post-exposure, the patient developed “severe headache, confusion, and aphasia, [and] generalized tonic-clonic seizures. Brain computed tomography hours after injection showed diffuse subarachnoid signal hyperattenuation. The patient fully recovered within 72 hours of injection.

An intraventricular catheter tubing was mistaken for an antecubital intravenous catheter in a 59-year-old man. The patient then experienced agitation, hypertension, and labile blood pressure shortly after receiving 10 mmol Magnevist (15). Magnetic resonance imaging of the patient’s ventriculostomy showed marked T1 shortening, confirming gadolinium presence. One day after exposure to gadolinium, the patient “developed new-onset aphasia, dysarthria, depressed mentation, a right facial droop, and increased urine output, suggesting diabetes insipidus.” One day following gadolinium administration, a head computed tomography scan showed “diffuse cerebral edema with crowding of basal cisterns and mild tonsillar descent.”

A 55-year-old woman developed altered mental status and severe headache one day after suspected intraventricular Magnevist administration (15). A head computed tomography scan showed “significant …hyperdense fluid within the dependent portions of the occipital horns of both lateral ventricles” and “diffuse cerebral edema remote from the operative site.” A non-contrast magnetic resonance imaging on day 1 showed signal enhancement within the patient’s basal cisterns and leptomeninges, and a susceptibility artifact was observed along the ventricular ependymal margins. The patient’s neurological status returned to baseline three days later.

Park et al. (16) documented a 42-year-old man who experienced confusion, global aphasia, and vomiting 6 hours after receiving an accidental intrathecal injection of 3 mmol Magnevist during a myelogram. Brain computed tomography imaging 10 hours after gadolinium administration showed diffuse subarachnoid hyperintensity. The stuporous patient was transferred to a neurosurgical intensive care unit, where he demonstrated severe rigidity, intermittent seizures, global aphasia, jerking movements of the left extremities, neck stiffness, 180/90 mmHg blood pressure, and 40.0℃ temperature. Angiography studies ruled out subarachnoid hemorrhage. Visual disturbances were documented four days later and persisted until day 7. The patient was discharged on the fifteenth day with no specific complaints. On 1-month follow-up, the patient noted recurrent visual disturbances. Bilateral optic atrophy persisted for one year.

Reeves et al. (17) reported a 60-year-old woman with iodine allergy who experienced “severe spastic pain with visible spasms of her lower extremities” approximately five minutes after receiving 2 mmol of Gadavist for intrathecal pain pump evaluation. She was ultimately discharged in stable condition and received follow-up at “4, 12, and 26 weeks [post-procedure] with no long-term sequelae.”

Singh et al. (18) described a 59-year-old man who underwent coil embolization of the right middle meningeal artery, resection of a tentorial meningioma, cranioplasty with titanium mesh, and external ventricular drain placement. Three days after the operation, the patient underwent a magnetic resonance imaging with 5 mmol Magnevist. The patient experienced nausea immediately following the magnetic resonance imaging and “became acutely hypertensive” within an hour. The images revealed diffuse intrathecal gadolinium, which was confirmed by imaging of the drain reservoir bag. By the following morning, he suffered “rapidly progressing aphasia, right facial droop, and delirium.” On the fifth day, his neurologic status continued to deteriorate. “Continuous electroencephalography revealed nonconvulsive status epilepticus.” For the following two months, the patient was comatose with punctuated seizures. Two years after the gadolinium exposure, the man resided “in a long-term facility, awake but not non-interactive or communicative with his environment.”

A 35-year-old woman developed encephalopathic symptoms, anaphylaxis, and disseminated intravascular coagulation after intravenous administration of 8 mmol gadobutrol for a brain magnetic resonance imaging scan (19). Immediately following the procedure, she experienced “sudden-onset shortness of breath, chest heaviness, diaphoresis, diarrhea, nausea, and vomiting.” Hypoxic respiratory distress warranted transfer to an emergency department. The patient was febrile (110.6°F), tachycardic (129 beats per minute), and hypotensive (68/43 mmHg) with a respiratory rate of 24 breaths per minute. She was intubated for respiratory distress with concomitant encephalopathy. Chemistries indicated respiratory and lactic acidosis and evolving acute kidney injury. Hypotension was refractory. While awaiting transfusion for disseminated intravascular coagulation, the patient succumbed to cardiac arrest following pulseless electrical activity. “Return of spontaneous circulation was achieved several times,” however, given the grim prognosis, the patient’s family opted for palliative measures. The patient had been treated both for suspected allergic reaction to gadolinium and possible sepsis, but blood cultures taken on intensive care admission returned showing no growth. The authors urged clinicians to be aware and alert regarding the possibility of allergic reactions with severe anaphylaxis, disseminated intravascular coagulation, and possibly fatal outcomes following gadolinium exposure.

# Supplementary Figures and Tables

**References**

1. Li L, Gao FQ, Zhang B, Luo BN, Yang ZY, Zhao J. Overdosage of intrathecal gadolinium and neurological response. Clin Radiol. 2008;63(9):1063-8.

2. Maramattom BV, Manno EM, Wijdicks EF, Lindell EP. Gadolinium encephalopathy in a patient with renal failure. Neurology. 2005;64(7):1276-8.

3. Provenzano DA, Pellis Z, DeRiggi L. Fatal gadolinium-induced encephalopathy following accidental intrathecal administration: a case report and a comprehensive evidence-based review. Reg Anesth Pain Med. 2019.

4. Samardzic D, Thamburaj K. Magnetic resonance characteristics and susceptibility weighted imaging of the brain in gadolinium encephalopathy. J Neuroimaging. 2015;25(1):136-9.

5. Besteher B, Chung HY, Mayer TE, Witte OW, Kirchhof K, Schwab M. Acute Encephalopathy and Cardiac Arrest Induced by Intrathecal Gadolinium Administration. Clin Neuroradiol. 2020;30(3):629-31.

6. Platt A, Ammar FE, Collins J, Ramos E, Goldenberg FD. Pseudo-subarachnoid hemorrhage and gadolinium encephalopathy following lumbar epidural steroid injection. Radiol Case Rep. 2020;15(10):1935-8.

7. Kapoor R, Liu J, Devasenapathy A, Gordin V. Gadolinium encephalopathy after intrathecal gadolinium injection. Pain Physician. 2010;13(5):E321-6.

8. Calvo N, Jamil M, Feldman S, Shah A, Nauman F, Ferrara J. Neurotoxicity from intrathecal gadolinium administration: Case presentation and brief review. Neurol Clin Pract. 2020;10(1):e7-e10.

9. Arlt S, Cepek L, Rustenbeck HH, Prange H, Reimers CD. Gadolinium encephalopathy due to accidental intrathecal administration of gadopentetate dimeglumine. J Neurol. 2007;254(6):810-2.

10. Moradian M, Tekmyster G, Wei JJ, Avetisian H, Acharya JN, Furman MB. Encephalopathy after unintentional intrathecal gadolinium: a letter to the editor. Interventional Pain Medicine. 2022;1(3):100105.

11. Pokersnik JA, Liu L, Simon EL. Contrast-induced encephalopathy presenting as acute subarachnoid hemorrhage. Am J Emerg Med. 2018;36(6):1122 e3- e4.

12. Sutherland E, Baird-Gunning J, Rudaks L, Palavra N, Lubomski M, Krause M. 075 Gadolinium encephalopathy presenting as status epilepticus following intrathecal injection. BMJ Neurology Open. 2021;3(Suppl 1):A27-A.

13. Palazón-Cabanes B, Martínez-García FA, Martínez-Lerma EJ, Hernández-Abenza J, Meca-Lallana JE, Martín-Fernández J. Is it safe intrathecal gadolinium administration? A new case of encephalopathy after performing an MR-cisternography. Revista Mexicana de Neurociencia. 2016;17(1):91-7.

14. Chauhan G, Upadhyay A. Gadolinium-based contrast agent-induced neurotoxicity: seeing is believing! BMJ Case Rep. 2021;14(1).

15. Nayak NB, Huang JC, Hathout GM, Shaba W, El-Saden SM. Complex imaging features of accidental cerebral intraventricular gadolinium administration. J Neurosurg. 2013;118(5):1130-4.

16. Park KW, Im SB, Kim BT, Hwang SC, Park JS, Shin WH. Neurotoxic manifestations of an overdose intrathecal injection of gadopentetate dimeglumine. J Korean Med Sci. 2010;25(3):505-8.

17. Reeves C, Galang E, Padalia R, Tran N, Padalia D. Intrathecal Injection of Gadobutrol: A Tale of Caution. J Pain Palliat Care Pharmacother. 2017;31(2):139-43.

18. Singh S, Rejai S, Antongiorgi Z, Gonzalez N, Stelzner M. Misconnections in the Critically Ill: Injection of High-Dose Gadolinium into an External Ventricular Drain. A A Case Rep. 2016;6(5):121-3.

19. Chalise SN, Palmer E, Pathak V. Fatal Allergic Reaction to Gadolinium Contrast. Cureus. 2023;15(7):e42455.
